# Effect of counseling model on diabetic women regarding sexual dysfunction: a quasi-experimental study

**DOI:** 10.1186/s12889-024-18585-9

**Published:** 2024-05-09

**Authors:** Nermen Awad Atia Abdelkhaliek, Soad Abdelsalam Ramadan, Seham Shehata Ibrahim, Maha Ramadan Ali Mohamed, Nour Elhoda Muhammad Elshabory

**Affiliations:** 1 Gynecology and Obstetrics Nursing, Faculty of Nursing, Port Said University, Port Said, Egypt; 2 Obstetric and Gynecological Nursing, Faculty of Nursing, Banha University, Banha, Egypt; 3 Gynecology and Obstetrics Nursing, Faculty of Nursing, Port Said University, Port Said, Egypt; 4 Gynecology and Obstetrics Nursing, Faculty of Nursing, Port Said University, Port Said, Egypt

**Keywords:** Counseling model, Diabetes, Female patients, Sexual dysfunction

## Abstract

**Background:**

Complications of diabetes in women have adverse effects on their self-image, quality of life, health, and other social relationships, thereby leading to sexual dysfunction. maternity nurse care can play a critical role in assessing the knowledge about needs for sexual health.

**Aim:**

The present study aims to evaluate the effect of the counseling model on female patients with diabetes regarding sexual dysfunction.

**Method:**

A quasi-experimental research design was used to conduct the study at the diabetic and obstetric outpatient clinic in 2 hospitals (Al Salam Port Said General Hospital, Elzohor General Hospital), and in five centers in Port Said City (El-Kuwait Center, Othman Ibnafan Center, El-arab 1 center, El-manakh center, El-arab2 center). A purposive sample of 178 female diabetic patients was included in the study. Two tools were used for collecting data consisted of; (1 interview questionnaire sheet) including personal characteristics, medical history, and present sexual problem of the studied female patients, (and 2 female sexual function index (FSFI).

**Results:**

the current study revealed that there was a high statistical difference between female sexual function in post with mean ± SD (23.3 ± 4.1) compared to pre-educational intervention with mean ± SD (19.5 ± 3.7), while there was a high statistically significant difference among pre- & post-program application regarding female sexual function index (*p* > 0.001).

**Conclusion:**

the counseling model had a positive effect in improving the sexual function among female patients with diabetes.

**Trial Registration Number (TRN):**

The study protocol was approved by the Research Ethics Committee of the Faculty of Nursing, Port Said University (code number: NUR 12/9/2021-6).

## Background

Sexual dysfunction is a common gynecological complaint among diabetic women due to its complicated effect on sexual function. Complications of diabetes in women have adverse effects on their self-image, quality of life, health, and other social relationships, thereby affecting their sexual performance [1]. Sexual dysfunction is a heterogeneous combination of disorders including abnormalities in women’s orgasm, arousal, pain, and unknown sexual dysfunction, although various studies have reported a high prevalence of sexual dysfunction in women with diabetes compared with non-diabetic women [2].

The prevalence of diabetes has risen significantly by 62% over the last ten years [3]. The International Diabetes Federation (IDF) listed Egypt among the world’s top 10 countries in the number of patients with diabetes [4] In Egypt, the prevalence of diabetes is around 15.56% among adults between 20 and 79 years of age, with an annual death of 86,478 related to diabetes [5].

Diabetes mellitus is a chronic metabolic disease characterized by insulin deficiency and resistance. The International Diabetes Federation (IDF) estimated that 7.5 million individuals have diabetes and around 2.2 million have prediabetes in Egypt. Furthermore, reports indicate that 43% of patients with diabetes and most patients with prediabetes in Egypt are likely undiagnosed. Consequently, the complications associated with the disease are also expected to increase. These include microvascular complications such as neuropathy, nephropathy, and retinopathy, and macrovascular complications such as peripheral artery disease, stroke, and cardiovascular diseases [6].

Diabetes is seen to be a risk factor for female sexual dysfunction because the normal female sexual response needs the integrity of the sensory and autonomic nervous system to respond to erotic stimuli, as well as of the vascular integrity which supplies the external genitalia and vagina which affected by hyperglycemia [7]. The main cause of sexual dysfunction in women with DM is multifactorial including biological, psychological, social, and interpersonal factors [8].

The PLISSIT model, which was described by Annon in 1974 for the first time consists of four main parts: (I) Permission (P), (II) Limited Information (LI), (III) Specific Suggestions (SS), (IV) Intensive Therapy (IT). By using the first three phases of the PLISSIT model, 80–90% of the patient’s sexual problems are solvable [9]. Education and counseling are effective in improving women’s sexual function through the provision of knowledge and problem-solving skills. Healthy lifestyles including nutrition, exercise, and sleep by controlling blood sugar levels protect a person from free radicals and indirectly affect their sexual function by regulating the body’s blood flow [10].

Maternity nurse care can play a critical role as a sex educator and sex counselor in this context. Also, she has an important role in assessing the knowledge about the needs for sexual health. Numerous frameworks are available for sexual advice that can help nurses implement appropriate and effective support strategies for intervention in the cases of sexual concerns and problems such as (ALARM, BETTER, PLEASURE, and PLISSIT). These models all take a somewhat different approach to sexual counseling [11].

Recently, evidence indicates that diabetic women are at higher risk for developing sexual dysfunction compared to those without diabetes [4]. There are several cultural and traditional hurdles to having open discussions about sexual life, especially with female health care practitioners, in Egypt, which makes it inappropriate to discuss female sexuality. Furthermore, female sexual dysfunction in diabetic patients is under-researched. It is frequently overlooked in research, with only a few studies addressing women’s sexual functioning and dysfunction in an Egyptian study [12]. Hence, this study has been conducted to improve the knowledge of female patients with diabetes regarding sexual dysfunction and be more able to solve the sexual dysfunction problem.

### Study aim

This study aims to evaluate the effect of the counseling model on female patients with diabetes regarding sexual dysfunction.

### Research hypothesis

#### Null hypothesis (H0)

there is no significant difference in the ability to resolve sexual dysfunction among diabetic women who receive counseling sessions compared to those who do not.

#### Alternative hypothesis (H1)

: diabetic women who receive counseling sessions demonstrate a greater ability to resolve sexual dysfunction compared to those who do not.

## Methods

### Research design

A Quasi-experimental research design (pre and post-test) was utilized to meet the aim of this study.

### Study setting

The current study was conducted at the diabetic and obstetric outpatient clinic in 2 hospitals (Al-Salam General Hospital, and El-zohor Central Hospital) and five centers affiliated the Health Insurance in Port Said City. These centers were selected randomly from twenty primary health care centers representing the five districts of Port Said according to the capacity of centers and registered cases namely (El-kwait Center, Othman Center, El-arab 1 Center, El-manakh Center, and El-arab2 Center).

### Study subjects

A purposive sample with total no. 178 women were included in the study according to the following criteria. A sample size of women was calculated according to the equation of **Daniel (1999).** Biostatistics: A foundation for Analysis in the Health Sciences. 7th edition. New York: John Wiley & Sons.

$$n = \frac{{{\rm{N}} \times {\rm{P}}\left( {1 - {\rm{P}}} \right)}}{{{\rm{N}} - 1 \times \left( {{{\rm{d}}^2} \div {z^2}} \right) + {\rm{P}}\left( {1 - {\rm{P}}} \right)}}$$

Where, N = total population (400); Z, Class standard corresponding to the level of significance equal to 0.95 and 1.96; D = error percentage (= 0.05); P = Ratio provides a neutral property = 0.50. Therefore,

$$n = \frac{{400 \times 0.5\left( {1 - 0.5} \right)}}{{(400 - 1) \times \left( {{{0.05}^2} \div 1.96} \right) + 0.5\left( {1 - 0.5} \right)}} = 162$$

The estimated sample size is 162, after adding the (10%) to avoid dropping out and/or incomplete responses or withdrawal, the final number for the sample size will be = 162 + 16 = 178.

### Inclusion criteria

- Married women who were diagnosed with diabetes with sexual dysfunction.
- Age 18–45 years.
- No pregnancy.
- No medical or obstetric disease except diabetes.

#### Tools for data collection

Two data collection instruments were used:

##### A structured interviewing questionnaire

The questionnaire was developed by the researcher in Arabic language. This tool includes three parts:

- **Part (I): sociodemographic characteristics of the female patients**: It includes data related to (age, educational level, occupation, family size, duration of marriage, residence, and body mass index).
- **Part (II): medical history**: It includes data related to (the duration of diabetes, type of diabetic treatment, and diabetic complications).
- **Part (III): Present sexual problem**: It includes data related to present sexual problems, its duration, circumcision, frequency of sexual intercourse, if getting help for sexual problems, and reason for not getting help.

##### Female sexual function index (FSFI)

This tool was developed by [12] to assess female sexual function. This scale consists of 19 items that are divided into 6 domains: [1] Desire 2 items [2], arousal 4 items [3], lubrication 4 items [4], orgasm 3 items [5], satisfaction 3 items and [6] pain 3 items. each domain was rated on a scale of 0 or 1–5 score, 0 indicates that the subject reports (having no sexual activity), 1 indicates (almost having no sexual activity), 2 indicates (a few times having sexual activity), 3 indicates (sometimes having sexual activity), 4 indicate (almost times having sexual activity) score 5 (suggestive of normal sexual activity).

### Tool validity

The face and content validity of the study tools were checked by a panel of seven experts consisting of (2 professors, 1 assistant professor, and 2 lectures from the maternity, Gynecology, and Obstetrics Nursing specialties). Professors reviewed the FSFI scale for Arabic language translation, clarity, relevance, comprehensiveness, and understanding applicability. The average proportion of Content Validity Index (CVI) for items judged relevant across the seven experts = 0.86. Comments and suggestions of the jury were considered and necessary modifications, corrections, and clarifying of the items were done accordingly.

### Tool reliability

The reliability of tools used in this study by the Cronbach’s alpha coefficient test to assess the internal consistency of the study tools. The internal consistency of the female sexual index was 0.91.

### Pilot study

A pilot study was conducted before starting the actual data collection. The pilot study was carried out on 10% (18 women) of the total sample of diabetic female patients. These were excluded from the main study sample. The purpose of the pilot study was to test the clarity, feasibility, and applicability of the study tools and estimate the time needed to complete the tools. It also helped to find out any obstacles and problems that might interfere with the data collection process. Needed modifications were done based on the findings of the pilot study.

### Ethical considerations

Approval was taken from the Research Ethics Committee of the Faculty of Nursing, Port Said University (code no. NUR 12/9/2021- 6). The purpose of the study was explained to the participants before obtaining the written consent to share in the study. A brief explanation of the study was given to assure the participants that all information obtained would be kept strictly confidential and used only for the study. Participants were informed that; they have the right to participate or withdraw from the study at any time. Code numbers instead of names of the participants were used for identification purposes. This measure ensured the participants would not be identified in the public reports.

### Fieldwork

The field of the study was conducted for eight months from the beginning of February 2022 to the end of December 2022. Data was collected 2 days a week, at the diabetic and obstetric outpatient clinic in 2 hospitals (Al Salam Port Said General Hospital, Elzohor General Hospital), and in five centers in Port Said City (El-Kuwait Center, Othman Ibnafan Center, El-arab 1 Center, El-manakh center, El-arab2 center), Average number of patients per week (6 patients). The study was carried out through the following phases:

#### Phase I (Assessment Phase)

in this stage, the researcher obtained official permission to carry out the study, the researcher visited the study settings and arranged with the nursing director for the actual implementation of the study. Then, the process of recruitment of the diabetic female patients according to the number of visits to the study settings. The researcher clarified the sheets of the two tools to each diabetic female patient and asked them to complete it in the waiting area. Each tool was filled in about 15 min to 30 min.

#### Phase II (Planning)

The counseling session for the participants was designed based on the baseline data collection. The session was conducted individually for each participant. The session aims to improve diabetic female patients’ sexual function of female patients with diabetes. The program was implemented in waiting areas in outpatient clinics by a researcher who had a master’s degree in obstetrics and gynecology and worked at a technical health institute and applied the session according to teaching strategies The counseling session was designed to cover information that contributes to success in managing diabetic illness and improving sexual function. The handout includes theoretical content and procedures for sexual dysfunction. Also, it was supplemented by photos and colors for more illustration and to help facilitate remembering knowledge. and covers the following content: introduction about diabetes, definition, risk factors, etiology, signs and symptoms, types of diabetes, the difference between hypoglycemia and hyperglycemia, complication, the effect of diabetes on sexual health, causes of relationship between diabetes and sexual health, healthy habits that increase women sexual health and uses of some herbs to enhance sexual health.

#### Phase III (The counseling session Implementation

In this phase counseling sessions based on the PLISSIT model were given on an individual basis, which include Permission (P), Limited Information (LI), Specific Suggestions (SS), and Intensive Therapy (IT). In the beginning, the researcher met with each diabetic female patient individually, explained the aim and procedures of the study, and invited them to participate. The patients who gave their informed consent to participate should also agree to provide their telephone numbers through which they could be contacted for follow-up. A copy of the handout was given to each patient to facilitate remembering the knowledge and practices during the explanation of the theoretical part.

The program was presented individually in clear and concise form using different teaching methods while discussing with them the rationale and the precaution for each step as small discussions, lectures, demonstrations, and re-demonstrations and appropriate teaching media as audiovisual material and real objects. At the end of the researcher’s demonstration patients were asked about any unclear steps which needed repetitions or explanation before demonstration. The researcher emphasized that this session was done for teaching purposes not for evaluation, so mistakes and forgetting were allowed and were corrected immediately by the researcher.

#### Phase IV (Evaluation phase)

The counseling session outcome was evaluated by using the second tool after the implementation phase for follow-up evaluation after three months. The researcher contacted patients through telephone numbers and WhatsApp applications.

### Statistical design

After completion of data collection, data was organized, tabulated, and computerized in Microsoft Excel 2021, and statically analyzed. The Statistical Package for Social Science (SPSS) version 28 was used to analyze the data on a PC. Data were presented using descriptive statistics in the form of frequencies and percentages for qualitative variables and means, and standard deviations for quantitative variables. Cronbach alpha coefficient was calculated to assess the reliability of the satisfaction scale through its internal consistency. Qualitative categorical variables were compared using the chi-square test. The obtained outcomes were considered significant at p-value ≤ 0.05 and highly significant at p-value ≤ 0.001 while p-value > 0.05 was considered non-significant.

## Results

### General characteristics of the studied female patients with diabetes

Table 1 shows, that 49.4% of the studied women were in the age group 29–38 years old, 47.8% of them had secondary or intermediate education, 87.6% of them had not worked and 77.3% of them were self–employed.

**Table 1:** General characteristics of the studied female patients with diabetes (*n* = 178)

General characteristics of female patients	*N*	%
**Age (Years)**		
20–28	23	12.9
29–38	88	49.4
39–45	67	37.6
**Mean ± SD**	37.3 ± 6.8	
**Educational level**		
Read and write	11	6.2
Basic education	72	40.4
Secondary or intermediate education	85	47.8
University qualification or higher	10	5.6
**Occupation**		
Working	22	12.4
Not working	156	87.6
**If the answer is yes, indicate the type of work (** ***n*** ** = 22)**		
Employee	5	22.7
Self– Employed	17	77.3

### General characteristics of the husband

Table 2 shows that 51.1% of the studied women’s husbands were in the age group 29–38 years old, and 41.0% of them had basic education, while 60.7% of husbands had self–employed and 82.6% of husb2-dMvGHxZWaza3:and had responsibility for income. Regarding the age of marriage, 52.2% of them were married at 20–30 years old, and 70.7% of the studied women had one marriage, 46.1% of them had duration of marital life between 11 and 15 years. Also, the table revealed that 65.7% of the studied women had from 3 to 4 children, while 64.6% of them mentioned number of residents in their house was 5–6 members, and 94.4% of them had 2–3 rooms in their house.

**Table 2:** General characteristics of the husband (*n* = 178)

General characteristics of the husband	*N*	%
**husband Age**		
20–28	10	5.6
29–38	91	51.1
39 or More	77	43.3
**husband educational level**		
Read and write	54	30.3
Basic education	73	41.0
Secondary or intermediate education	29	16.3
University qualification or higher	22	12.4
**husband Occupation**		
Employee	66	37.1
Pension	4	2.2
Self– Employed	108	60.7
**Family income**		
Enough	44	24.7
Not enough	99	55.6
It is sufficient and overflowing	35	19.7
**Responsibility for Income**		
Husband	147	82.6
Both	31	17.4
**The age of marriage**		
Less than 20	85	47.7
20–30	93	52.2
**The number of marriages**		
One	126	70.7
Two	52	29.2
**Duration of marital life (Years)**		
Less than 5	28	15.7
5–10	18	10.1
11–15	82	46.1
More than 15	50	28.1
**Number of children**		
1–2	53	29.8
3–4	117	65.7
More than 4	8	4.5
**Number of residents in the house**		
3–4	54	30.3
5–6	115	64.6
More than 6	9	5.1
**Number of rooms in the house**		
One room	3	1.7
2–3 rooms	168	94.4
More than 3	7	3.9

### Distribution of the studied female patients with diabetes according to their medical history

Table 3 shows that 48.3% of the studied women started their diabetes illness from one year to 5 years, 74.2% of them made regular examinations of diabetes, and 92.7% of them had type I Diabetes, 62% of them had regular diabetes. Also, 64.6% of them used insulin in treatment, and 78.7% of them have complications from diabetes and 76.4% of them suffer from chronic diseases, 55.6% of them take other medication.

**Table 3:** Distribution of the studied female patients with diabetes according to their medical history (*n* = 178)

medical history	*N*	%
**When did your diabetes start?**		
Less than a year	46	25.8
From one year to 5 years	86	48.3
More than 5 years	46	25.8
**Examination of diabetes**		
Regular	132	74.2
Irregular	46	25.8
**Type of diabetes**		
Diabetes type I	165	92.7
Diabetes mellitus type II	13	7.3
**Regularity of diabetes**		
Regular	111	62.4
Irregular	67	37.6
**Type of treatment**		
Insulin	115	64.6
Discs	58	32.6
Diet	5	2.8
**Are there complications from diabetes?**		
Yes	140	78.7
No	38	21.3
**Do you suffer from chronic diseases?**		
Yes	42	23.6
No	136	76.4
**Do you take any other medications?**		
Yes	99	55.6
No	79	44.4

### Distribution of the studied female patients with diabetes according to their pregnancy history

Table 4 illustrates that, 70.8% of the studied women were pregnant 2–3 times, 68.5% of them had a range 2–3 number of births, 56.2% of them had from 1 to 3 living children and 71.9% of them were delivered by cesarian section.

**Table 4:** Distribution of the studied female patients with diabetes according to their pregnancy history (*n* = 178)

pregnancy history	*N*	%
**The number of pregnancies**		
Once	17	9.6
2–3	126	70.8
More than 3	35	19.7
**The number of births**		
Once	11	6.2
2–3	122	68.5
More than 3	45	25.3
**Number of abortions**		
None	46	25.8
Once	36	20.2
Twice	81	45.5
More than twice	15	8.4
**Number of living children**		
None	9	5.1
1–3	100	56.2
More than 3	69	38.7
**Method of delivery**		
Normal	50	28.1
Cesarian	128	71.9

### Distribution of the studied female patients with diabetes according to their sexual history

Table 5 presents that, 86.0% of the studied women had no problems in sexual relations, while 36.0% of women who mentioned they had problems clarified that the problem started since than one year. Also, 60.7% of the studied women suffer from sexually transmitted diseases, and 54.5% of them have performed circumcision. The current table also illustrates that 50.6% of women had irregular intercourse time, 63.5% of them didn’t take drugs that help with sexual intercourse and 57.9% of them turn to their family when they have sexual problems.

**Table 5:** Distribution of the studied female patients with diabetes according to their sexual history (*n* = 178)

sexual history	*N*	%
**Are there any current problems in sexual relations?**		
Yes	25	14.0
No	153	86.0
**If yes, when did this problem start? (** ***n*** ** = 25)**		
Less than a year	9	36.0
From one year to 3 years	8	32.0
More than 3 years	8	32.0
**Do you suffer from sexually transmitted diseases?**		
Yes	70	39.3
No	108	60.7
**Was circumcision performed?**		
Yes	81	45.5
No	97	54.5
**Number of intercourse times**		
Regular	88	49.4
Irregular	90	50.6
**Do you take drugs that help sexual intercourse?**		
Yes	65	36.5
No	113	63.5
**Whom do you turn to for help when you have sexual problems?**		
Medical assistance	69	38.8
The family	103	57.9
Friends	6	3.4

### Relation between the female sexual function index at pre and post-counseling sessions

Table 6 there was a high statistical difference between female sexual function in post with mean ± SD (23.3 ± 4.1) compared to pre-educational intervention with mean ± SD 19.5 ± 3.7.

**Table 6:** Relation between the female sexual function index at pre and post-counseling sessions (*n* = 178)

	Pre– Intervention	Post Intervention	Student’s T–Test	
	Mean ± SD	Mean ± SD	T	*P*
Desire	3.2 ± 0.8	3.9 ± 0.9	7.755	< 0.001**
Arousal	3.3 ± 0.7	3.9 ± 0.8	7.530	< 0.001**
Lubrication	3.3 ± 0.8	3.9 ± 0.8	7.075	< 0.001**
Orgasm	3.3 ± 0.7	3.9 ± 0.7	8.086	< 0.001**
Satisfaction	3.2 ± 0.6	3.9 ± 0.9	8.634	< 0.001**
Pain	3.2 ± 0.7	3.8 ± 0.8	7.530	< 0.001**
**Total Score**	**19.5 ± 3.7**	**23.3 ± 4.1**	**12.189**	< 0.001**

### The female sexual function index at pre and post-counseling sessions

**Fig. 1:**
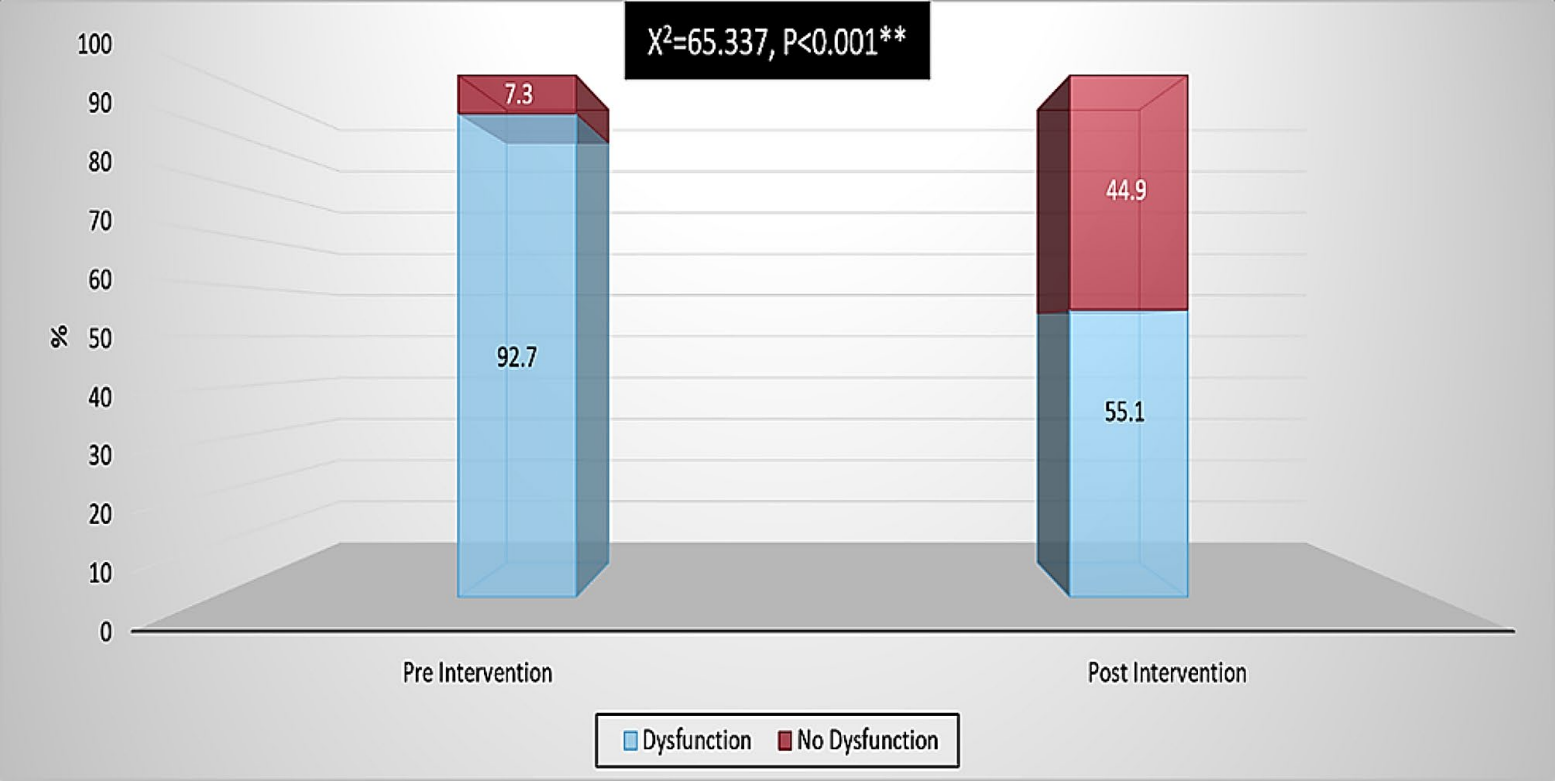
The female sexual function index at pre and post-counseling sessions

Figure (1) presents that there were high statistically significant difference notices among pre & post-program applications regarding the female sexual function index (*p* > 0.001**).

### Relation between the female medical history at pre and post-counseling sessions

Table 7 there was no statistical relation between female sexual function in pre and post-program with their medical history.

**Table 7:** Relation between the female medical history and female sexual index pre and post counseling sessions (*n* = 178)

	Pre– Intervention		Post– Intervention	
	Mean ± SD	Significance test	Mean ± SD	Significance test
**When did your diabetes start?**				
Less than a year	19.06 ± 3.76	F = 1.305, *P* = 0.274	22.38 ± 3.96	F = 1.097, *P* = 0.336
From one year to 5 years	19.29 ± 3.63		22.20 ± 4.14	
More than 5 years	20.20 ± 3.68		21.23 ± 4.31	
**Examination of diabetes**				
Regular	19.32 ± 3.65	T = 0.869, *P* = 0.386	21.71 ± 4.17	T = 1.534, *P* = 0.127
Irregular	19.87 ± 3.77		22.80 ± 3.98	
**Type of diabetes**				
Diabetes type I	19.53 ± 3.72	T = 0.835, *P* = 0.405	21.84 ± 4.18	T = 1.826, *P* = 0.070
Diabetes mellitus type II	18.64 ± 3.08		24.00 ± 2.97	
**Regularity of diabetes**				
Regular	19.08 ± 3.67	T = 1.815, *P* = 0.071	21.98 ± 4.11	T = 0.052, *P* = 0.959
Irregular	20.11 ± 3.64		22.02 ± 4.22	
**Type of treatment**				
Insulin	19.01 ± 3.64	F = 2.567, *P* = 0.080	22.07 ± 4.11	F = 0.649, *P* = 0.524
Discs	20.32 ± 3.69		21.70 ± 4.25	
Diet	20.12 ± 3.39		23.82 ± 3.94	
**Are there complications from diabetes?**				
Yes	19.08 ± 3.74	T = 0.730, *P* = 0.467	21.62 ± 3.95	T = 0.621, *P* = 0.535
No	19.57 ± 3.67		22.10 ± 4.20	
**Do you suffer from chronic diseases?**				
Yes	19.21 ± 3.62	T = 0.507, *P* = 0.613	22.06 ± 4.09	T = 0.115, *P* = 0.909
No	19.54 ± 3.71		21.98 ± 4.17	
**Do you take any other medications?**				
Yes	19.86 ± 3.97	T = 1.597, *P* = 0.112	21.79 ± 4.21	T = 0.723, *P* = 0.470
No	18.97 ± 3.25		22.25 ± 4.07	

## Discussion

A normal sex life is an important part of life and relationships. Diabetes mellitus (DM) is an important cause of sexual dysfunction both in men and women. This problem is more difficult to diagnose and treat in women than in men because of the intricacy of the female sexual response. Also, the literature is limited in addressing female sexual dysfunction (FSD) in DM, and this aspect of female health is often ignored in clinical practice in women with DM. Early screening, diagnosis, and appropriate counseling are the cornerstone for managing FSD in women with DM [13]. So, it was very necessary to shed light on this sensitive problem through the present research using the PLISSIT model to facilitate data collection & counseling to overcome the negative consequences of that problem [14]. The present study aimed to evaluate the effect of the sexual counseling model on the sexual dysfunction of women with diabetes & their sexual quality of life.

Regarding the medical history of the studied female patients, the present study shows that more than two-fifths of women with diabetes started from one year to five years, and more than two-thirds of them make regular examinations for diabetes. Regarding the type of diabetes, the majority of the women had type I Diabetes, and more than half of them had regular diabetes. Concerning the treatment of diabetes more than half of them used insulin in treatment, most of them have complications from diabetes and most of them suffered from chronic diseases. These results were supported by [15] who studied the effect of the counseling model on sexual dysfunction among women with diabetes and their sexual quality of life in Minia, and mentioned that more than one-third of the studied sample had diabetes for less than 10 years & slightly three-quarters of diabetic women had type 1 diabetes mellitus who treated by insulin only. In Egypt [16] concluded that the prevalence of sexual dysfunction is higher in type one diabetic women, compared to type two diabetes type one is more common to appear before the age of 40 years.

Regarding to pregnancy history of the studied female patients, the findings showed that most of the women had two to three times of pregnancies and more than two-thirds of them had two to three births number. Moreover, regarding the number of living children more than half of them have one to three children and most of them are cesarian. These results agreed with Gerges et al. [10] who studied sexual dysfunction in women with diabetes in Banha and revealed that more than two-thirds of the studied women had from one to three children, in contrast to the result of the current study more than two-thirds of them were delivered with Vaginal delivery mode.

Regarding the sexual history of the studied female patients, the present findings presented that the majority of the sample hadn’t problems in sexual relationships, concerning those who have sexual problems more than one-third of them mentioned the problem started since than one year ago. Regarding circumcision, more than half performed circumcision, and more than half had irregular intercourse time. This was disagreed with Ayalew [17]; Kirici, and Emel et al. [18] who revealed that sexual relation problems were correlated significantly with diabetes among the studied women. Concerning circumcision the present result agreed with Abd-elatief, Mohasib, and Mohamed [15]; and Arafa et al. [16] who reported that the majority of the sample were circumcised.

Concerning female sexual function index (FSFI) domains; the present findings confirmed that diabetic women had a significant improvement in all domains post-educational intervention compared to pre-educational intervention. This result was the same line with Abd-elatief, Mohasib & Mohamed [15] who reported that after 6 months of an educational program diabetic women had the highest mean score related to their female sexual function index domains & total mean scores of female sexual functions had a significant improvement from 23.8 pre-education to 29.9 after six months of education with P– ≤ 0.000. In the researcher point view it might be related to the lack of knowledge among women about sexual function and also regarding the fact that in Egypt the culture of the society it was shy and non-ethical to that woman for discussing this kind of knowledge. Another study by Mehrabi, Lotfi, Rahimzadeh, & Khoei [19], on 100 married women aged 35–55 years old with type 2 diabetes, reported that PLISSIT model-based sexual counseling increased all domains of sexual function, except for sexual excitement and pain.

## Conclusion

### Based on the findings of the present study, it can be concluded that

Counseling by using the PLASSIT model has affected positively female sexual function outcomes which manifested by improving the score of the female sexual function index after the implementation of the counseling model.

#### Implications

This can be done by Increasing women’s awareness about factors affecting sexual health and teaching them how to manage and encourage them to seek help & discuss their sexual concerns with healthcare providers. make educational programs based on the PLISSIT model for diabetic women in all available diabetic clinics or centers to improve women’s sexual function. Adopting sexual counseling based on the PLISSIT model in addressing sexual dysfunction for diabetic women with the Preparation of a secure environment in the hospital outpatient units to discuss sexual problems with women freely. Also, can Integrate the concept of sexual counseling based on the PLISSIT model in addressing sexual dysfunctions among diabetic women into undergraduate curricula of faculties of nursing and in-service educational programs for caregivers on how to deal and manage various sexual dysfunctions for diabetic women by using PLISSIT counseling model. Sexual problems should be included in the Ministry of Health and Population plan to care for diabetic women with sexual dysfunctions using the PLISSIT counseling model.

### Limitation of the study

It was difficult and took more time to meet patients who met the inclusion criteria and more time to collect data and give them the counseling session individually. It was also difficult to collect information about sexual dysfunction because the patients felt embarrassed to talk about this sensitive subject.

## Data Availability

The datasets used and/or analysed during the current study available from the corresponding author on reasonable request.
